# TOmicsVis: An all‐in‐one transcriptomic analysis and visualization R package with Shinyapp interface

**DOI:** 10.1002/imt2.137

**Published:** 2023-10-13

**Authors:** Ben‐Ben Miao, Wei Dong, Zhao‐Fang Han, Xuan Luo, Cai‐Huan Ke, Wei‐Wei You

**Affiliations:** ^1^ State Key Laboratory of Marine Environmental Science, College of Ocean and Earth Sciences Xiamen University Xiamen China; ^2^ Hospital of Stomatology, Guanghua School of Stomatology, Guangdong Provincial Key Laboratory of Stomatology Sun Yat‐Sen University Guangzhou China; ^3^ Fujian Institute for Sustainable Oceans Xiamen University Xiamen China

**Keywords:** data visualization, differential expression, enrichment analysis, multigroup RNA‐Seq, shinyapp

## Abstract

Transcriptomic analysis has been widely used in comparative experiments to uncover biological mechanisms in various species. However, a simple tool is still lacking to optimize and integrate the features from multiple R packages. In this study, we developed TOmicsVis (Transcriptomics Visualization) (CRAN: https://cran.r-project.org/package=TOmicsVis, v2.0.0), an R package that provides a comprehensive solution for transcriptomics analysis and visualization. It utilizes 46 R packages to design 40 suitable functions for the streamlined analysis of multigroup transcriptomic projects, which covers six main categories: Sample Statistics, Traits Analysis, Differential Expression, Advanced Analysis, GO and KEGG Enrichment, and Table Operation. TOmicsVis can be performed either locally or online (https://shiny.hiplot.cn/tomicsvis-shiny/), which provides significant convenience for researchers without coding training. These user‐friendly visualization functions and built‐in analysis capabilities enable researchers to monitor experimental data dynamics promptly and explore transcriptomics data quickly.

## Highlights

- Transcriptomics Visualization (TOmicsVis) package provides a one‐stop analysis and visualization pipeline for two or more groups of transcriptomics projects, with multigrouped example data sets.
- TOmicsVis offers a local run function for Shinyapp and an online interface analysis service, providing significant convenience for researchers without coding abilities.
- TOmicsVis integrates advanced analysis and visualization methods such as gene expression trend analysis, gene clustering analysis, and weighted gene co‐expression network analysis.

## Graphical abstract

Transcriptomics Visualization (TOmicsVis) implements 40 functions for transcriptomics data analytics and visualization based on 46 R packages, which provide a one‐stop analysis and visualization pipeline for two or more groups of transcriptomics projects, with multigrouped example data sets. TOmicsVis offers a local run function for Shinyapp and an online interface analysis service (https://shiny.hiplot.cn/tomicsvis-shiny/), providing significant convenience for researchers without coding skills.

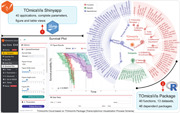

## INTRODUCTION

In the past decade, transcriptomics has become a powerful quantitative method for studying gene expression, playing a crucial role in biological research [1]. By comparing gene expression types and abundance under different experimental conditions, researchers can identify numerous differentially expressed genes (DEGs) that are closely linked to specific traits. Transcriptomics has a wide range of applications, allowing for the investigation of gene expression during various growth stages and the examination of the impact of biological or environmental factors on gene expression in organisms [2]. Furthermore, transcriptomics research in non‐model species does not necessarily require a reference genome, enabling the discovery of gene expression profiles and biological features across a wider range of species. The application of transcriptomics in multiple species has been accelerated by next‐generation sequencing (NGS) technologies led by Illumina and third‐generation sequencing (TGS) technologies led by PacBio. The continuous advancements in sequencing technologies, combined with the use of genomes and the iterative improvement of transcriptomic analysis, have led to the rapid development and updating of analysis tools in this field.

Transcriptome sequencing produces FastQ files that contain quality information and require a series of steps for transcript quantification and analysis. Typically, this involves sequence filtering using a program such as fastp [3] and genome alignment using a program such as HISAT2 [4]. For transcriptome analysis without a reference genome, the Trinity program [5] is recommended for transcript assembly. Once gene expression levels are normalized across samples, DESeq2 package [6] is utilized to identify DEGs among groups. Venn and volcano diagrams are commonly used to visualize the number and distribution of DEGs. Gene Ontology (GO) [7] and Kyoto Encyclopedia of Genes and Genomes (KEGG) [8] annotation and enrichment analysis are crucial in exploring gene functions, and clusterProfiler [9] is a popular method for enrichment analysis based on the hypergeometric distribution algorithm. Weighted Gene Co‐expression Network Analysis (WGCNA) [10] involves constructing co‐expression modules based on all transcripts and is applied to identify gene modules that are highly correlated with the studied traits. There are numerous R packages available for analyzing and visualizing different steps in transcriptomics. Hence, there is a pressing need for comprehensive integration and optimization.

The Transcriptomics Visualization (TOmicsVis) R package is designed to integrate conventional analysis and deep mining methods to provide a one‐stop analysis and visualization solution for transcriptome projects. In transcriptomic downstream analysis, a variety of advanced analysis methods are built in addition to differential expression analysis. Meanwhile, TOmicsVis offers alternatives for each step, such as the dimensionality reduction analysis section with principal component analysis (PCA), t‐Distribution Random Neighborhood Embedding (t‐SNE), and Uniform Manifold Approximation and Projection (UMAP) algorithms for producing tabular and image results. TOmicsVis Shinyapp provides complete interface functions, which can help researchers perform transcriptomic data analytics and visualization work smoothly and efficiently.

## RESULTS AND DISCUSSIONS

### Overview categories and visualization functions

**Figure 1:**
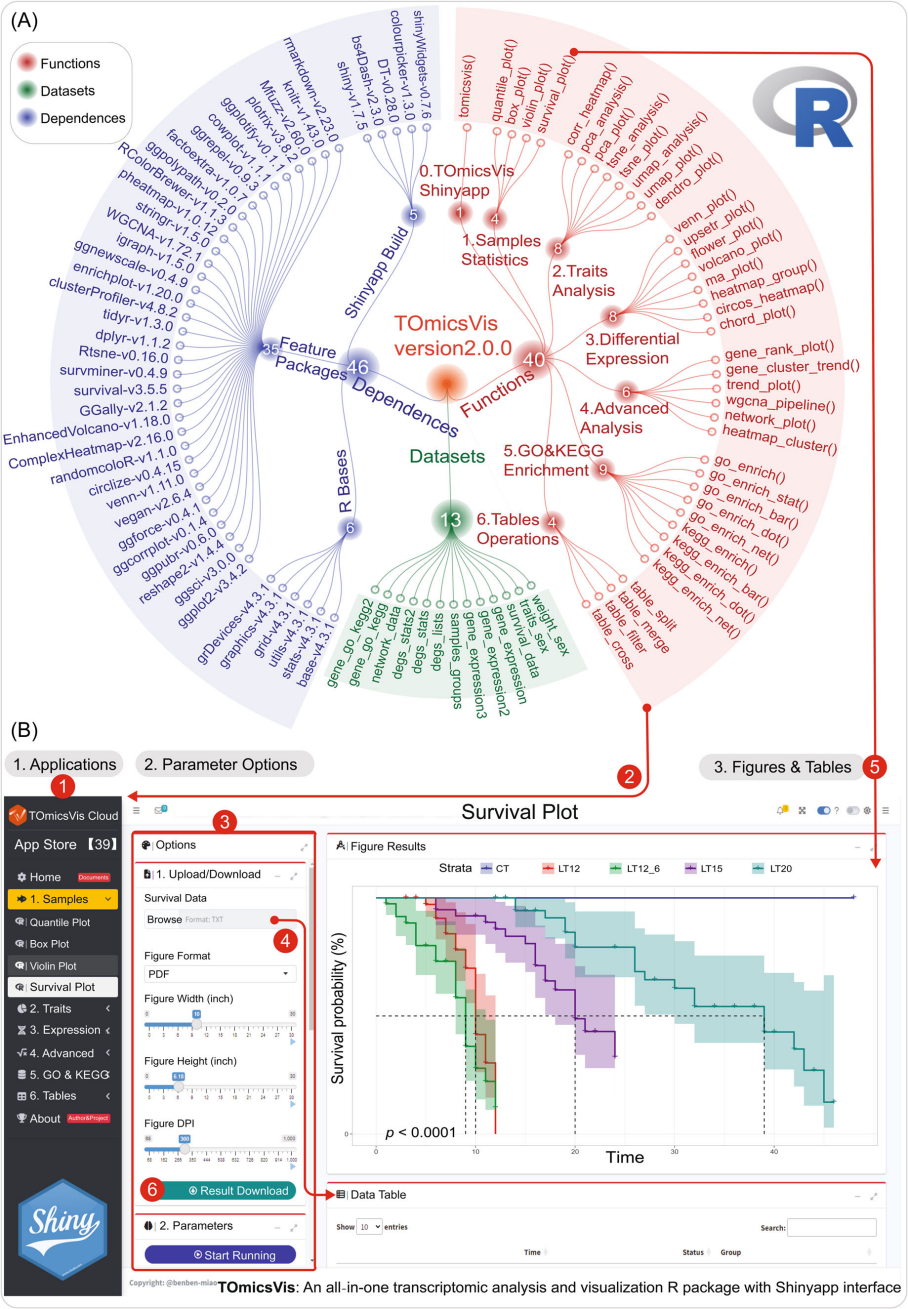
The logical architecture of Transcriptomics Visualization (TOmicsVis) R package and TOmicsVis Shinyapp. (A) TOmicsVis implements 40 functions for transcriptomics data visualization based on 46 R packages, and built‐in 13 data sets. Visualization functions are highlighted in red, dependent packages in blue, and built‐in example data sets in green. (B) TOmicsVis Shinyapp user interface. The numbers 1‐6 marked with a red background indicate the execute steps of Shinyapp: 1. Open the sub‐application; 2. Call the functions; 3. Switch to the parameter panel; 4. Browse input data; 5. Execute the survival_plot function in the example; and 6. Download the PDF result file. GO, Gene Ontology; KEGG, Kyoto Encyclopedia of Genes and Genomes.

TOmicsVis relies on a total of 46 R packages, including six basic packages [11], five packages for building Shinyapp, and the remaining 35 third‐party packages for performing data processing, transcriptome analysis, and visualization customization. Based on these dependent packages, TOmicsVis implements 40 functions for data analytics and visualization, covering the entire process of transcriptome projects from sample traits to gene expression analysis. These categories include Samples Statistics, Traits Analysis, Differential Expression, Advanced Analysis, GO and KEGG Enrichment, and Table Operation. There is a special function TOmicsVis to start our Shinyapp interface system, specially customized for the TOmicsVis R package. Based on published transcriptomics research articles with multiple groups [12], we have prepared 13 example data sets built into TOmicsVis package. These data sets are referred to as function example codes, and it is convenient for users to quickly prepare data (Figure 1A).

The functions in the Samples Statistics category are used to perform statistics and test differences among measurable traits and to perform survival analysis on environmental stress survival records. The Traits Analysis category including correlation analysis based on Pearson or Spearman methods, dimensionality reduction analysis based on PCA, t‐SNE, UMAP algorithms, and hierarchical agglomerative clustering analysis using Ward's method. In the Differential Expression category, Venn, upset, heatmap, circle heatmap, and chord diagrams are used to visualize gene expression differences among multiple groups. Volcano and M‐versus‐A (MA) diagrams are suitable for displaying DEGs between two paired groups. Advanced transcriptome analysis takes all genes into account and expands deep data mining beyond differential expression analysis. For instance, gene_cluster_trend function first performs cluster analysis on all genes in all groups and then builds multiple gene profiles with consistent expression trends. wgcna_pipeline first performs cluster analysis on all genes and then builds co‐expression modules that are closely related to the traits studied based on the WGCNA v1.72.1 [10] package. The functions in the GO and KEGG Enrichment category have been rewritten based on the clusterProfiler v4.8.2 [9] package. We normalize GO and KEGG annotation tables based on reference and non‐reference genomes and generate new functions from data processing and enrichment analysis.

### TOmicsVis Shinyapp practicality

To make the TOmicsVis functions more user‐friendly for researchers without coding skills, we have developed a user‐friendly interactive analysis and visualization interface platform based on shiny v1.7.5 (https://github.com/rstudio/shiny/), bs4Dash v2.3.0 (https://github.com/RinteRface/bs4Dash), and so forth packages. The tomicsvis function allows users to start and deploy locally after installing TOmicsVis package, at which point all computations will use local computer resources, including memory, CPU, and storage. More conveniently, we offer free online services and computing resources for researchers to access and perform analytical tasks anytime, anywhere (https://shiny.hiplot.cn/tomicsvis-shiny/).

The TOmicsVis Shinyapp user interface features an application menu sidebar, a parameter operation panel, and a figure and table display panel from left to right (Figure 1B). The Home and About page on the application menu displays the TOmicsVis API documentation and project information, respectively. The remaining six folding menus contain applications corresponding to all functions by category. TOmicsVis Shinyapp is written in 18,000+ lines of R code and enables the complete analysis and visualization of all 40 functions. We have marked the order of the program running steps. Upon clicking to start an application, the parameter operation panel and the figure and table display panel will switch accordingly. The parameter operation panel is divided into two sections: data upload and download parameters, and analysis and visualization parameters from top to bottom. Thanks to the various file, text, value, options, colors, and button web components provided by the Shiny framework, we provide specific interactive interfaces for all the parameters of the function. The final results can be viewed in the display panels, the figure result can be downloaded in PDF or JPEG format (default DPI value is 300), and the table results can be downloaded in TXT format. In conclusion, the fast implementation and stable operation of the TOmicsVis Shinyapp are aided by the out‐of‐the‐box functions of TOmicsVis, which complement each other for long‐term maintenance and in‐depth development work.

### TOmicsVis resources and competitiveness

TOmicsVis is open source under GENERAL PUBLIC LICENSE (GPL) license, and all source codes of R package, API documentation, websites, and Shinyapp are stored in the GitHub repository https://github.com/benben-miao/TOmicsVis. TOmicsVis provides a stable version for multi‐platform installation on the Comprehensive R Archive Network (CRAN) website (https://cran.r-project.org/package=TOmicsVis), and the latest unstable version is available on the GitHub repository. Furthermore, we have created comprehensive API documentation and tutorials, including text and video content (https://benben-miao.github.io/TOmicsVis/). TOmicsVis Shinyapp (https://shiny.hiplot.cn/tomicsvis-shiny/) will be maintained for a long time to provide stable online analysis services to users. As a result, after a month since its release, TOmicsVis has gained significant traction with over 700+ unique installations (by the date of 2023‐09‐13).

To clarify the benefits of TOmicsVis, we compared its capabilities and resources with several other commonly used R packages and web servers (Table 1). The advantage of the R packages systemPipeR v2.6.3 [13], RNASeqR v1.0.3 [14], and bcbioRNASeq v0.5.5 [15] is that it includes the whole process of transcriptome analysis based on raw reads. Compared with them, TOmicsVis focuses more on downstream data analytics and extreme visualization and is suitable for multigroup transcriptome projects. TOmicsVis includes algorithm‐based gene expression clustering and trend analysis methods, providing a variety of candidate gene deep mining methods and visualization in addition to differential expression analysis. Compared with Hiplot [16] and ImageGP [17] bioinformatics web servers, TOmicsVis Shinyapp, the user‐friendly and interactive web interface of TOmicsVis, is designed to provide real‐time cloud computing services. Its unique feature is that as an open‐source R package, it can continually receive more optimization and be called by other packages to enhance its contribution to transcriptome analysis. In conclusion, the goal of TOmicsVis is to create an optimized R package that provides an interface analysis platform based on these robust functions.

**Table 1:** Comprehensive comparison of TOmicsVis with three R packages and two websites.

Comment Points	TOmicsVis	systemPipeR	RNASeqR	bcbioRNASeq	Hiplot	ImageGP
Current version	v2.0.0	v2.6.3 [13]	v1.0.3 [14]	v0.5.5 [15]	v2023 [16]	v2023 [17]
R Package/website	Package and website	Package and website	Package	Package	Website	Website
Code writing	Optional	Optional	Need	Need	No need	No need
Shiny function	Yes	Yes	No	No	Null	Null
Raw reads start	No	Yes	Yes	Yes	Yes	No
Data visualization	Yes	Yes	Yes	Yes	Yes	Yes
Extreme visualize	Yes	No	No	No	Yes	Yes
Samples and traits	Yes	No	No	No	Yes	Yes
RNA‐Seq pipeline	Downstream	Whole process	Whole process	Whole process	Downstream	Downstream
Deep mining	Yes	No	No	No	Yes	No
Clusters and trends	Yes	No	No	No	Yes	No
Innovative way	Optimization and interface	Pipeline and interface	Pipeline and integration	Pipeline and reports	Multi‐omics interface	Bio‐data visualization
Long‐term maintenance	Yes	Yes	Yes	Yes	Yes	Yes
Link address (source code)	https://github.com/benben-miao/TOmicsVis/	https://github.com/tgirke/systemPipeR/	https://github.com/HowardChao/RNASeqR/	https://github.com/hbc/bcbioRNASeq/	https://hiplot.cn/	https://www.bic.ac.cn/ImageGP/index.php

### User cases of TOmicsVis

#### Case 1: Samples statistics and traits analysis in experiments

To demonstrate the effectiveness of TOmicsVis in multigroup transcriptomics research projects, we conducted an analysis of data from published research articles in the User Case section [12].

**Figure 2:**
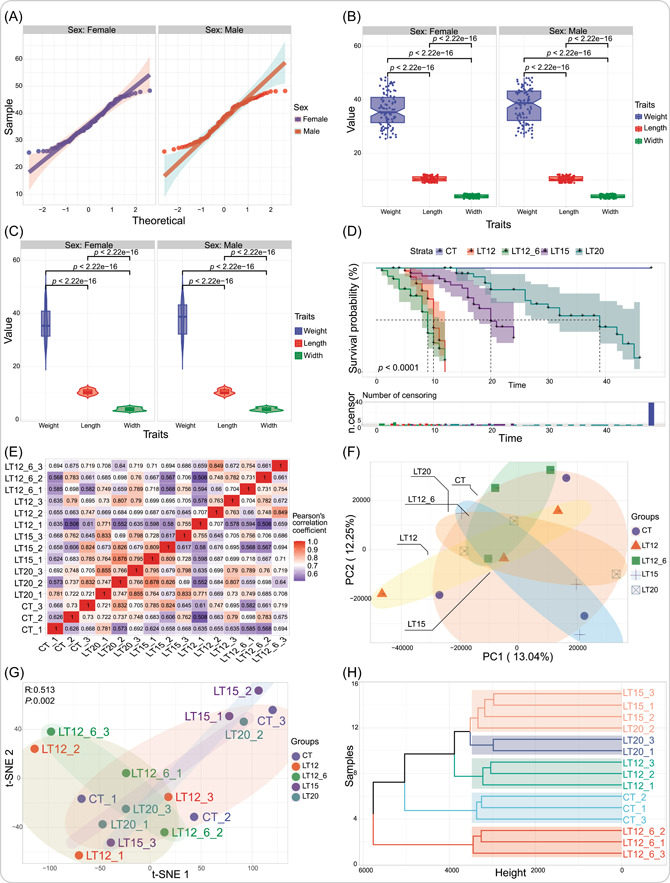
Sample statistics and trait analysis implemented in Transcriptomics Visualization (TOmicsVis). (A) Quantile plot. (B) Box plot. (C) Violin plot. (D) Survival analysis plot. (E) Correlation heatmap. (F) Principal component analysis (PCA) analysis plot. The 95% confidential interval is indicated on the plot. (G) t‐Distribution Random Neighborhood Embedding (t‐SNE) analysis plot. The *p*‐value and R value are shown on the plot. (H) Hierarchical clustering dendrogram. These results were obtained using TOmicsVis to analyze real transcriptome project data containing five groups, which are also used as built‐in data sets. Element sizes and color spectra in the images can be customized by modifying the parameters of the functions.

After field or laboratory sampling is completed, a normal distribution test is performed on the weights of all 200 samples using the quantile_plot function with a 95% confidence interval set. This function enables users to control the integration or separation of multiple groups for display (Figure 2A). Subsequently, statistical analysis and differential testing need to be performed on multiple measurable traits. At this point, both the box_plot and violin_plot are viable options. Both of them support annotating significant *p* value in the resulting graphs and comparing groups. There were significant statistical differences (*p* value < 0.05) between paired traits in the results (Figure 2B,C). For experiments involving extreme environmental stress or toxicity testing, the survival_plot function can effectively analyze and interpret the recorded data of sample survival over time. The results of this study showed that the lower the temperature stress, the lower the survival rate, while sample survival was not affected under normal room temperature (Figure 2D). To summarize, TOmicsVis emphasizes the preparation of measurable data during the experimental process and fully considers the complexity of the data and the practical needs of researchers in the analysis and visualization of results.

To analyze the correlations among all samples within and between groups, a corr_heatmap is used to perform Pearson, Spearman, or Kendall correlation coefficients for all gene expression data and display them in a heatmap. Its uniqueness lies in the fact that the median often corresponds to extreme color values, allowing users to display correlation coefficients with only positive or negative values. The analysis results show that samples within groups exhibit higher correlations than those among groups (Figure 2E). Dimensionality reduction algorithms are used to uncover the distribution relationships among samples based on a large number of genes. The gene expression matrix in this demonstration consists of 11,033 genes, corresponding to 15 samples from five groups. The PCA dimensionality reduction algorithm is widely used, and we provide two functions, pca_analysis and pca_plot, which generate analysis results tables and diagrams, respectively. The function allows users to specify any two PC values for display and add ellipsoidal backgrounds representing 95% confidence intervals for each group (Figure 2F). Furthermore, t‐SNE and UMAP algorithms are recommended for use in SingleCell workflow, so the analysis and visualization functions for these two algorithms are included in TOmicsVis (Figure 2G). Clustering algorithms are still effective for classifying samples. The dendro_plot function first performs a cluster analysis and displays the hierarchical evolutionary tree (rectangle, circular, or phylogenetic) of the distance relationships among samples. It defaults to using Euclidean or Canberra distance methods and Ward's hierarchical agglomerative cluster algorithm. The results reveal sample clustering distance trees that nearly align with the experimental sampling scheme (Figure 2H). To summarize, TOmicsVis integrates multiple algorithms such as Pearson's, PCA, t‐SNE, UMAP, Euclidean, and Ward's hierarchical agglomerative cluster to explore grouping traits from the perspectives of correlation, dimensionality reduction, and clustering.

#### Case 2: Screening candidate genes by multigroup differential expression analysis

In transcriptome research, the identification and interpretation of DEGs are the main focus of the analysis process. The biological functions of DEGs help us understand the impact of experimental conditions on gene transcription levels and biological processes within the organism, based on their functions.

**Figure 3:**
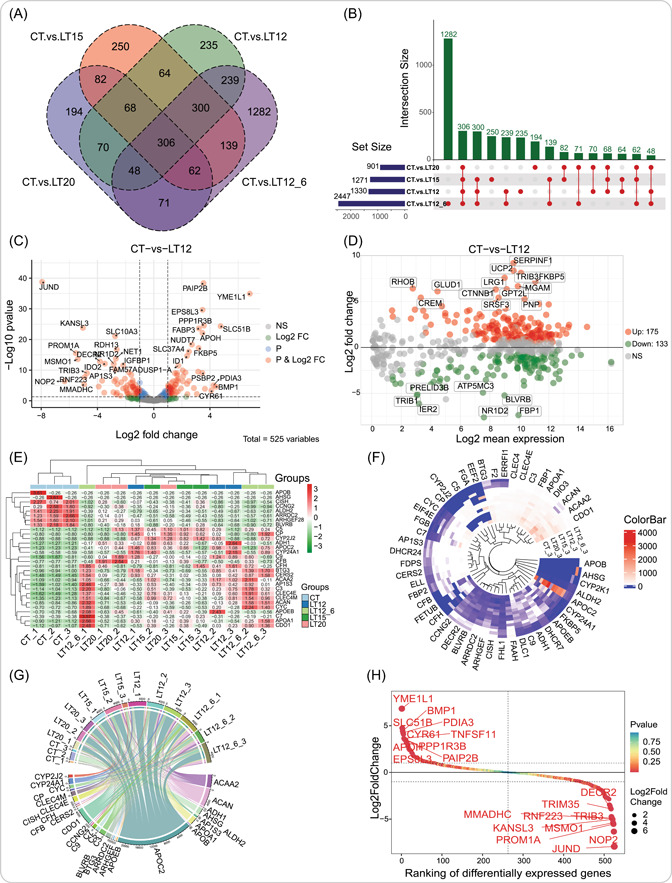
Differential expression analysis and visualization of genes among groups. (A) Venn diagram. (B) Multiintersection upSet diagram. (C) Volcanic scatter plot. (D) M‐versus‐A plot. (E) Multigroup clustering heatmap. (F) Circle heatmap. (G) Chord plot. (H) Genes ranking plot. These results were obtained using Transcriptomics Visualization (TOmicsVis) to analyze real transcriptome project data containing five groups, which are also used as built‐in data sets. Element sizes and color spectra in the images can be customized by modifying the parameters of the functions.

Based on five groups, we are conducting differential expression analysis between the CT group and the other four low‐temperature stress experimental groups. After identifying DEGs in these four pairwise comparisons, the venn_plot function be used to visualize the unique and shared DEGs among these comparisons. This function allows for a maximum of seven data intersections among comparisons. The results show that the CT.versusLT12_6 comparison has the highest number of DEGs, indicating that lower temperatures and longer exposure to stress lead to more genes exhibiting differential expression. There is a total of 306 differentially expressed genes shared among all four comparisons (Figure 3A). Given the need for data intersection analysis when dealing with multiple groups, the upsetr_plot function can overcome the limitations of the venn_plot (Figure 3B). After analyzing the statistical results of differential expression analysis across all comparisons, it becomes possible to identify more crucial genes in pairwise comparisons. The volcano_plot function uses the default criteria of |Log2FoldChange | > 1 and *p* value ≤ 0.05 (equal:‐Log10Pvalue ≥ 1.30103) for genes screening. Genes that meet these criteria are represented by red dots, while blue, green, and gray dots represent genes that only meet the *p* value, Log2FoldChange, or do not meet the filtering conditions, respectively. Part important DEGs in the results are labeled with gene symbols for the convenience of researchers to locate and focus on (Figure 3C). The M‐versus‐A visualization is carried out by the ma_plot function, which is similar to the volcano_plot in terms of its purpose to display and annotate DEGs (Figure 3D).

Based on the 306 shared DEGs that were screened, we conducted further analysis to explore gene expression variations across all samples. Actually, we only selected the top 30 genes for demonstration purposes. The heatmap_group function initially performs a clustering analysis on all 15 samples and presents the results of sample clustering and gene expression in the form of a tree and a heatmap, respectively. This function allows users to adjust the orientation of axis titles, heatmap cell coloring, and additional color variations for all groups (Figure 3E). When more genes need to be displayed, rectangular heatmaps are limited by space, so the circos_heatmap can be used as a complementary method to heatmap_group. Clearly, in the results shown in Figure 3F, 50 DEGs are displayed and the gene names are still clearly visible. Interestingly, we displayed the gene expression values as the width of the chords in the chord_plot, which effectively displays the expression characteristics of 30 DEGs across all 15 samples. As a result, the DEGs *CDO1*, *APOC2*, *ACAN*, and *ACAA2* are worth monitoring (Figure 3G). After a series of differential expression analyses, genes with the highest expression change are not negligible. The gene_rank_plot function ranks all DEGs based on Log2FoldChange and screens the top‐10 genes with the highest fold changes in both upregulation and downregulation for visualization (this value is adjustable). As a result, DEGs like *YME1L1* and *JUND* require further validation (Figure 3H).

#### Case 3: Mining gene clusters by constructing co‐expression modules and trends

Transcriptomics advanced analysis aims to uncover more potential gene expression profiles, especially in experiments involving multiple groups (≥3 groups). Two main approaches are currently exploring gene expression trends and constructing gene clusters through clustering analysis. Genes with similar expression characteristics are analyzed together, which provides potential biological insights.

**Figure 4:**
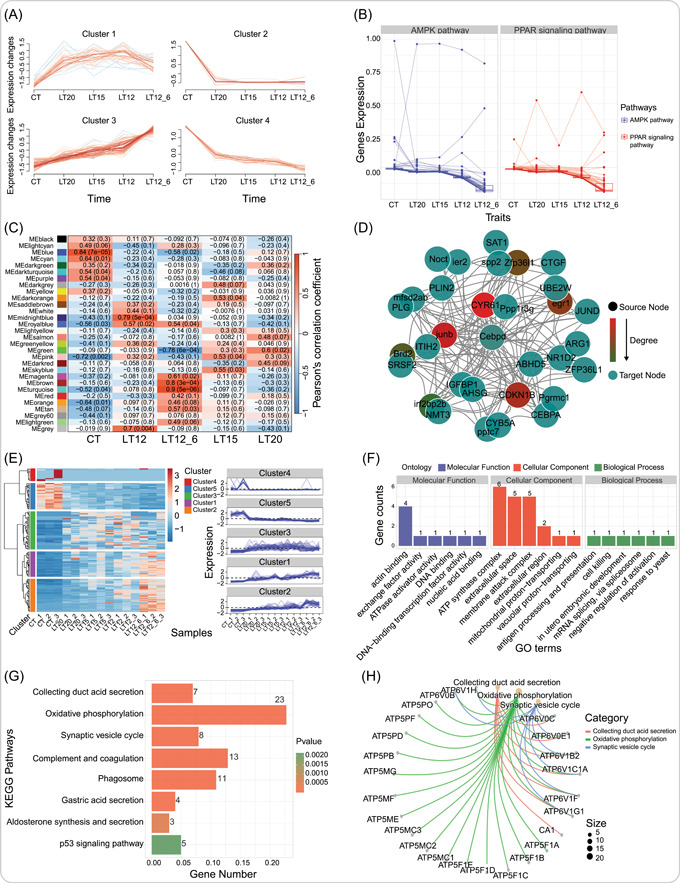
Advanced transcriptomic analyses included in the TOmicsVis. (A) Genes clustering and trends. (B) Gene expression trends. (C) Modules and traits correlation heatmap. (D) Interaction network among genes. (E) Gene clustering heatmap and trend integration. (F) GO enrichment statistics plot. (G) KEGG enrichment bar chart. (H) KEGG enrichment network graph. These results were obtained using Transcriptomics Visualization (TOmicsVis) to analyze real transcriptome project data containing five groups, which are also used as built‐in data sets. Element sizes and color spectra in the images can be customized by modifying the parameters of the functions. GO, Gene Ontology; KEGG, Kyoto Encyclopedia of Genes and Genomes.

The gene_cluster_trend function provides a comprehensive analysis and visualization function based on a series of functions provided by the Mfuzz v2.60.0 [18] package, such as creating expression sets based on gene expression matrix, data normalization, executing clustering algorithms, and visualizing trend plots. Four clusters were automatically constructed in the results, and the genes in each cluster displayed similar expression patterns across all groups, with genes in Cluster 1 and Cluster 3 upregulated and genes in Cluster 2 and Cluster 4 downregulated (Figure 4A). The trend_plot function does not perform data clustering analysis but instead normalizes the data to explore gene expression trends among groups. Normalization methods include std (standard), globalminmax (global minimum and maximum values), centerObs (center observes), and so forth. It also supports faceting based on KEGG pathways (Figure 4B).

We have created a primary analysis pipeline based on the official documentation of the WGCNA package, including data preparation and preprocessing, screening optimal soft thresholds for constructing co‐expression modules, analyzing correlations among modules, and correlations between modules and traits. Eventually, I will be producing several key tables and figure results. Among the module‐trait correlation heatmap results, genes in the MEmidnightblue module (*R* = 0.79, *p* = 5e^−4^) and the MEgrey module (*R* = 0.7, *p* = 0.004) are highly correlated with the LT12 group. The genes in the MEturquoise module (*R* = 0.9, *p* = 5e^−6^) and the MEbrown module (*R* = 0.8, *p* = 3e^−4^) are highly correlated with the LT12_6 group (Figure 4C). The network_plot function was utilized to construct an interaction network among the top‐200 gene pairs obtained from the WGCNA follow‐up analysis. Compared to the Cytoscape v3.10.1 [19] program, network_plot calculates the interaction frequency based on inbound and outbound connectivity and provides a variety of network structures. The results default to the sphere structure display, where *CYR61*, *CEBPD*, and *PPP1R3G* all belong to the hub genes and are closely related to other genes (Figure 4D). The heatmap_cluster function combines clustering and trend analysis, allowing the user to specify the number of clusters. In the results, we have created five clusters, with genes in Cluster 1, Cluster 2, and Cluster 3 showing upregulated expression, and Cluster 3 has the highest number of genes (Figure 4E).

Gene annotation and enrichment analysis are crucial methods for obtaining gene biological function information. Utilizing the clusterProfiler package to perform enrichment analysis based on the hypergeometric distribution algorithm is convenient, especially with annotation information from multiple model species. In actual transcriptome projects, there are two additional ways to obtain GO and KEGG annotation data: (a) Annotation information from a new or private reference genome that is not included in clusterProfiler. (b) For species without a reference genome, transcripts are assembled, and annotation information must be obtained through BLAST against the GO and KEGG databases. Ultimately, we standardized the GO and KEGG annotation tables obtained through various methods and redesigned functions for enrich analysis and visualization. Provide researchers with tables and graphical results to enrich the analysis. The go_enrich_stat function performs GO enrichment analysis and statistical visualization (Figure 4F). The kegg_enrich_bar and kegg_enrich_net functions, respectively, output bar chart and network graph based on KEGG enrichment analysis (Figure 4G,H).

## CONCLUSION

TOmicsVis package uses 46R packages to design 40 functions suitable for the streamlined analysis of multigroup transcriptomics projects and provide 13 RNA‐Seq example data sets. These functions are divided into six categories: Sample Statistics, Traits Analysis, Differential Expression, Advanced Analysis, GO and KEGG Enrichment, and Table Operation, effectively covering the needs of conventional transcriptomics project analysis. The TOmicsVis R package is maintained on CRAN (https://cran.r-project.org/package=TOmicsVis), accumulating a unique download count of 700+ times (by the date of 2023‐09‐13). Additionally, it offers a local version of the Shinyapp and an online analysis service (https://shiny.hiplot.cn/tomicsvis-shiny/), which provides researchers with significant convenience without coding abilities.

## METHODS

### TOmicsVis built‐in data sets for process demonstration

TOmicsVis built‐in complete transcriptomics example data sets allow for testing the function and parameter details at any time. These data sets are compiled from published transcriptomics research articles [12], which include 25°C room temperature (CT), 20°C, 15°C, 12°C, and 12°C hypothermic stress for 6‐h groups (LT20, LT15, LT12, and LT12_6); each group contains three replicates for a total of 15 samples. This study can effectively test the benefits of TOmicsVis in multigroup transcriptomics studies, and the experimental data, including only two groups, remains applicable. We standardize according to the actual situation by using samples, traits, gene expression data, and so forth, so users can quickly access it in experiments (Table 2). Among them, weight_sex, traits_sex, and survival_data contain information on the measured traits of the samples, and gene_expression, gene_expression2, gene_expression3, and samples_groups contain gene expression data for all samples. degs_lists, degs_stats, and degs_stats2 contain statistical data of DEGs; gene_go_kegg and gene_go_kegg2 contain GO and KEGG annotation information of genes; and network_data contain interaction data of genes. These data sets will be installed with the TOmicsVis R package, and these tabular data can be downloaded from the source code repository (https://github.com/benben-miao/TOmicsVis/tree/main/inst/data-tables/).

**Table 2:** Transcriptomics data sets built into the TOmicsVis package.

Data name	Description	Functions
weight_sex	Weight and sex traits data frame.	quantile_plot
traits_sex	Length, width, weight, and sex traits data frame.	box_plot, violin_plot, table_filter
survival_data	Survival records data.	survival_plot
gene_expression	Expression data frame for all genes in all samples in RNA‐Seq.	corr_heatmap, pca_analysis, pca_plot, tsne_analysis, tsne_plot, umap_analysis, umap_plot, dendro_plot, wgcna_pipeline
samples_groups	Attribution among samples and groups.	pca_analysis, pca_plot, tsne_analysis, tsne_plot, umap_analysis, umap_plot
degs_lists	DEGs lists of paired comparisons among groups.	venn_plot, upsetr_plot, flower_plot
degs_stats	Differential statistics of all DEGs of CT‐vs‐LT12 comparison.	volcano_plot, gene_rank_plot
degs_stats2	Differential statistics of all DEGs of CT‐vs‐LT12 comparison.	ma_plot
gene_expression2	Expression data of shared DEGs of all paired comparisons.	heatmap_group, circos_heatmap, chord_plot, heatmap_cluster, table_cross
gene_expression3	Expression data and pathway of shared DEGs of all comparisons.	gene_cluster_trend, trend_plot
network_data	Network data from WGCNA tan module top‐200 data frame.	network_plot
gene_go_kegg	GO and KEGG annotation of all genes in RNA‐Seq.	go_enrich, go_enrich_stat, go_enrich_bar, go_enrich_dot, go_enrich_net, kegg_enrich, kegg_enrich_bar, kegg_enrich_dot, kegg_enrich_net, table_cross
gene_go_kegg2	GO and KEGG annotation of all genes in RNA‐Seq.	table_split, table_merge

*Note*: All these data sets are the default data for the visualization functions, which can be found at https://github.com/benben-miao/TOmicsVis/tree/main/inst/data-tables/.

### Infrastructure for development

TOmicsVis is developed using the R v4.3.1 [11] and utilizes the roxygen2 v7.2.3 package for generating and updating API documentation. The correctness of functions and the integrity of the package are verified using the devtools v2.4.5 package. The compilation is performed based on the DESCRIPTION and NAMESPACE files. The API documentation and website are built using pkgdown v2.0.7 with the _pkgdown.yml configuration file, which provides online help documentation (https://benben-miao.github.io/TOmicsVis/). For data manipulation, the dplyr v1.1.2, and tidyr v1.3.0 packages from the tidyverse ecosystem are used to transform data structures. TOmicsVis prioritizes the use of ggplot2 v3.4.2, a widely used package that offers customizable visualization capabilities [20]. Additionally, the ggsci v3.0.0 package is utilized to provide publication‐friendly discrete color palettes.

## AUTHOR CONTRIBUTIONS

Ben‐Ben Miao conceived and designed the project. Ben‐Ben Miao and Wei Dong wrote and tested the R codes of the TOmicsVis package. Zhao‐Fang Han, Xuan Luo, Cai‐Huan Ke, and Wei‐Wei You provided funding and support for the project. Zhao‐Fang Han and Wei‐Wei You provided scientific and development suggestions for the project. Ben‐Ben Miao prepared the figures and tables and wrote the manuscript. Wei Dong and Wei‐Wei You reviewed and revised the manuscript. All authors read and approved the final manuscript.

## CONFLICT OF INTEREST STATEMENT

The authors declare no conflict of interest.

## Data Availability

The source code of TOmicsVis R package, Shinyapp, and example data sets on GitHub repository: https://github.com/benben-miao/TOmicsVis/, API document and tutorials website: https://benben-miao.github.io/TOmicsVis/, TOmicsVis on CRAN: https://cran.r-project.org/package=TOmicsVis, Online Shinyapp service: https://shiny.hiplot.cn/tomicsvis-shiny/. Supplementary materials (graphical abstract, slides, videos, Chinese translated version, and update materials) may be found in the online DOI or iMeta Science http://www.imeta.science/.
